# Clinical characteristics and diagnoses of 1213 children referred to a first seizure clinic

**DOI:** 10.1002/epi4.12883

**Published:** 2024-01-25

**Authors:** Geertruida Slinger, Lotte Noorlag, Eric van Diessen, Willem M. Otte, Maeike Zijlmans, Floor E. Jansen, Kees P. J. Braun

**Affiliations:** ^1^ Department of Neurology and Neurosurgery, UMC Utrecht Brain Center University Medical Center Utrecht and Utrecht University Utrecht The Netherlands; ^2^ Department of Pediatrics, Franciscus Gasthuis & Vlietland Rotterdam The Netherlands; ^3^ Stichting Epilepsie Instellingen Nederland (SEIN) Heemstede The Netherlands

**Keywords:** epidemiology, epilepsy diagnosis, first seizure, first seizure clinic, pediatric epilepsy

## Abstract

**Objective:**

New‐onset seizure‐like events (SLEs) are common in children, but differentiating between epilepsy and its mimics is challenging. This study provides an overview of the clinical characteristics, diagnoses, and corresponding etiologies of children evaluated at a first seizure clinic (FSC), which will be helpful for all physicians involved in the care of children with SLEs.

**Methods:**

We included 1213 children who were referred to the FSC of a Dutch tertiary children's hospital over a 13‐year period and described their clinical characteristics, first routine EEG recording results, and the distribution and specification of their eventual epilepsy and non‐epilepsy diagnoses. The time interval to correct diagnosis and the diagnostic accuracy of the FSC were evaluated.

**Results:**

“Epilepsy” was eventually diagnosed in 407 children (33.5%), “no epilepsy” in 737 (60.8%), and the diagnosis remained “unclear” in 69 (5.7%). Epileptiform abnormalities were seen in 60.9% of the EEG recordings in the “epilepsy” group, and in 5.7% and 11.6% of the “no epilepsy” and “unclear” group, respectively. Of all children with final “epilepsy” and “no epilepsy” diagnoses, 68.6% already received their diagnosis at FSC consultation, and 2.9% of the children were initially misdiagnosed. The mean time to final diagnosis was 2.0 months, and 91.3% of all children received their final diagnosis within 12 months after the FSC consultation.

**Significance:**

We describe the largest pediatric FSC cohort to date, which can serve as a clinical frame of reference. The experience and expertise built at FSCs will improve and accelerate diagnosis in children with SLEs.

**Plain language summary:**

Many children experience events that resemble but not necessarily are seizures. Distinguishing between seizures and seizure mimics is important but challenging. Specialized first‐seizure clinics can help with this. Here, we report data from 1213 children who were referred to the first seizure clinic of a Dutch children's hospital. One‐third of them were diagnosed with epilepsy. In 68.8% of all children—with and without epilepsy—the diagnosis was made during the first consultation. Less than 3% were misdiagnosed. This study may help physicians in what to expect regarding the diagnoses in children who present with events that resemble seizures.

**Key points**

- New‐onset seizure‐like events are common, particularly in children, but differentiating epilepsy from its mimics is challenging.
- First seizure clinics may improve and accelerate diagnosis. In our cohort, 68.6% received their diagnosis at first consultation, and less than 3% were misdiagnosed.
- This study can serve as a reference framework for the distribution and specification of diagnoses in children with new‐onset seizure‐like events.

## 1 INTRODUCTION

Physicians often face a diagnostic challenge when people present with new‐onset seizure‐like events (SLEs). This challenge particularly holds true in children, in whom the incidence of epilepsy is relatively high,^1^ clinical symptoms are diverse, and epilepsy mimics occur frequently.^2^ Consequently, false‐positive and false‐negative epilepsy diagnoses in children are common, with reported misdiagnosis rates up to 35%.^3^, ^4^, ^5^, ^6^

Timely and accurate differentiation between epilepsy and its mimics is crucial and will determine management, prognosis, and socioemotional impact. Electroencephalography (EEG) is a commonly used and valuable tool in the diagnostic workup of SLEs. Nevertheless, the first routine EEG recording is often normal in children with epilepsy^7^, ^8^, ^9^ and, conversely, may show epileptiform abnormalities in children without epilepsy.^10^ Therefore, epilepsy largely remains a clinical diagnosis requiring experience and expertise. In the last two decades, hospitals have established dedicated pediatric first seizure clinics (FSCs) where this expertise is provided.^6^, ^11^, ^12^, ^13^ Their first results are promising, indicating that these clinics may reduce waiting times for the initial evaluation^11^ and contribute to a timely diagnosis and proper management.^12^, ^13^ A comprehensive overview of diagnoses and the diagnostic accuracy in a large, multi‐year pediatric FSC cohort could serve as a clinical frame of reference for the distribution and specification of diagnoses in children with new‐onset SLEs but is currently lacking.

We present data from more than 1200 children who were referred to the FSC of a Dutch tertiary children's hospital over a 13‐year period. We describe the children's clinical characteristics, their diagnosis, and corresponding etiologies. In addition, we summarize the results of the first routine EEG recording and evaluate the time interval to correct diagnosis and the diagnostic accuracy of our FSC.

## 2 METHODS

### 2.1 Study setting

Our FSC was established on January 1, 2008 to provide fast and integrated care to children with new‐onset SLEs. The one‐day FSC consultation includes an in‐depth history taking, neurological examination, and routine EEG recording, including activation procedures. The results are discussed the same day in a team with the participation of a pediatric neurologist, clinical neurophysiologist, neurology resident, and epilepsy nurse. They together decide on a diagnosis and, if needed, a treatment plan or further diagnostic workup. Epilepsy diagnoses are made according to the International League Against Epilepsy definition of epilepsy.^14^ Children with epilepsy often remain under our outpatient care for follow‐up. Children without epilepsy are usually referred back to their referrer, with the note to return to our clinic in case the frequency or semiology of the events changes or when a second (unprovoked) seizure occurs after a single seizure.

### 2.2 Patient selection

We retrospectively included all children (0–18 years) who were referred to the FSC of the Wilhelmina Children's Hospital, Utrecht, the Netherlands between January 1, 2008, and May 31, 2021, after experiencing one or more new‐onset SLEs. Data were collected between June 2021 and May 2022. We maintained a time interval of at least 1 year between the FSC consultation and data collection to ensure sufficient time for the diagnostic workup. The institutional ethics committee approved the use of anonymized retrospective data for research purposes without informed consent (protocol numbers 09‐353/K and 18‐354/C).

### 2.3 Data collection

All data were obtained via medical record auditing. Definitions of all variables are given in Table S1.

#### 2.3.1 Demographics and medical history

Demographic data included sex, age at first SLE and FSC consultation, and referral data. Medical history data included family history, history of epilepsy or seizures, neurological history, presence of any neurodevelopmental disorders, developmental concerns, intellectual disability, and genetic syndromes.

#### 2.3.2 Diagnosis

For each child, we extracted the initial FSC diagnosis (i.e., the diagnosis made at the end of the FSC consultation), the final diagnosis (i.e., the diagnosis at the latest follow‐up, if required after additional investigations), the etiological diagnosis (i.e., the cause of the “epilepsy” or the “no epilepsy” diagnosis), and the time interval to final and etiological diagnosis.

Initial diagnostic options were: “epilepsy,” “single unprovoked seizure” (SUS), “no epilepsy,” or “unclear.” Final diagnostic options were: “epilepsy,” “no epilepsy,” or “unclear.” If the correct diagnosis was made at the FSC, the time to final diagnosis was set to 0. In other cases, the time to diagnosis was calculated as the interval between the FSC date and the date the epilepsy diagnosis was confirmed or rejected. A SUS was eventually categorized as either “epilepsy” or “no epilepsy,” based on the results of additional investigations or the occurrence of a second unprovoked seizure in the 12 months following the first seizure. If children did not return with a second seizure or event and no correspondence from other hospitals regarding a second seizure or event was received, we assumed these had not occurred. The initial diagnosis was considered “unclear” if additional investigations were deemed necessary to confirm or reject the epilepsy diagnosis. The final diagnosis was “unclear” if doubts persisted about the origin of the SLEs, even after additional investigations (Table S2).

We specified etiological diagnoses for “epilepsy” and “no epilepsy.” Epilepsy type, syndrome, and etiology diagnoses were made according to the ILAE guidelines.^14^, ^15^, ^16^ Between 2008 and 2021, epilepsy syndromes were diagnosed according to older ILAE definitions, but their names have been converted to the most recent ones for the purpose of this study. “No epilepsy” etiological diagnoses were classified into nine categories: provoked seizures (both febrile and acute symptomatic seizures), cardiovascular, respiratory, or behavioral events, psychological or psychiatric conditions, sleep‐related events, paroxysmal movement disorders, migraine‐associated disorders, and miscellaneous.^2^

#### 2.3.3 FSC EEG recording

We classified the results of the FSC EEG recording as normal or abnormal, based on the clinical neurophysiologist's report. Abnormalities were categorized as aspecific, focal epileptiform, and generalized epileptiform. We extracted whether or not the SLEs were captured during the EEG recording.

#### 2.3.4 Follow‐up

We reviewed children's medical records until their latest retrievable outpatient clinic visit or phone consultation. For children with epilepsy, we extracted if they were on antiseizure medication (ASM) or other epilepsy treatments at the last follow‐up, and if they underwent epilepsy surgery. We defined seizure freedom at the last follow‐up according to *Kwan* et al. (2010): “freedom from seizures for a minimum of three times the longest preintervention interseizure interval (determined from seizures occurring within the past 12 months) or 12 months, whichever is longer.”^17^

### 2.4 Data analysis

We used descriptive statistics to summarize clinical characteristics. Group comparisons were performed with nonparametric Wilcoxon‐Mann–Whitney tests for continuous data and Fisher's exact tests for categorical data. Categorical data are presented as frequencies and percentages and continuous data as medians and ranges unless otherwise specified. All analyses were performed with R statistical software, version 4.3.0.^18^

## 3 RESULTS

### 3.1 Demographics

Between January 1, 2008, and May 31, 2021, 1292 children were evaluated at our FSC. We excluded 71 children with an established epilepsy diagnosis before the FSC consultation and eight without SLEs. Eventually, 1213 children (55.1% boys) were included.

Median age at FSC consultation was 5.8 (0.1–17.7) years. Data on the children's medical history are presented in Table 1 and Table S3. Most children entered the FSC via their general practitioner (42.8%) or internal referral (36.9%). The remaining children were referred by an external healthcare worker (18.5%), or came in via self‐referral (1.1%) or an unknown route (0.7%). Of the children referred by an internal or external healthcare worker, 243 (36.2%) had been seen at the emergency department before referral to the FSC. Based on 1185 observations, the median time from referral to FSC consultation was 22 (1–236) days.

**TABLE 1:** Study population characteristics.

Characteristic^a^	Total (*N* = 1213)
Demographics	
Sex, boy	668 (55.1)
Age at FSLE	4.2 (0–17.6)^b^
Age at FSC consultation	5.8 (0.1–17.7)
Family history^c^ ^,^ ^d^ ^,^ ^e^	
Epilepsy	245 (20.4)
Febrile seizures	130 (10.8)
Migraine	134 (11.2)
Other	100 (8.3)
Medical history^d^	
Epilepsy	28 (2.3)
Seizures^e^	163 (13.4)
Typical febrile seizures	101 (8.3)
Atypical febrile seizures	34 (2.8)
Neonatal convulsions	38 (3.1)
Other	15 (1.2)
Neurological^e^	233 (19.2)
Perinatal asphyxia	30 (2.5)
Hemorrhage or stroke	66 (5.4)
CNS infection	18 (1.5)
Head trauma	64 (5.3)
Migraine	11 (0.9)
Other	95 (7.8)
Neurodevelopmental disorders^e^	146 (12.0)
AD(H)D	59 (4.9)
ASD	106 (8.7)
Genetic syndrome or condition	98 (8.1)
Developmental status	
Developmental concerns at presentation^f^	426 (35.1)
Intellectual disability^g^	181 (14.9)

Abbreviations: AD(H)D, attention deficit (hyperactivity) disorder; ASD: autism spectrum disorder; CNS: central nervous system; FSLE, first seizure‐like event; FSC, first seizure clinic.

^a^ N (%) for categorical variables, and median (range) for continuous variables.

^b^ Data based on 1087 observations, because of an unknown age at FSLE for 126 children.

^c^ Family history in first‐ and second‐degree relatives; data based on 1201 observations, because of an unknown family history for 12 children.

^d^ Known at the time of the FSC consultation.

^e^ Subcategories of family history, seizures, neurological history, and neurodevelopmental disorders are not mutually exclusive.

^f^ Any, either being cognitive, motor, language/speech, social, emotional, or behavioral delay, mentioned by parent(s) or attending physician.

^g^ Established or estimated IQ <70, either known at the time of the FSC consultation or during follow‐up.

### 3.2 Distribution of final diagnoses

Of the 1213 children, 407 (33.5%) were eventually diagnosed with “epilepsy,” 737 (60.8%) received a “no epilepsy” final diagnosis, and the diagnosis remained “unclear” in 69 children (5.7%) (Table 2). Age‐stratified analyses showed that adolescents most and neonates and infants least frequently received an epilepsy diagnosis (45.1% versus 16.8%) (Table S4A). The distribution of final diagnoses was roughly comparable between referrers (Table S5).

**TABLE 2:** Specified final diagnoses.

Diagnosis^a^	Total (*N* = 1213)
Epilepsy	407 (33.5)
Epilepsy type	
Focal	254 (62.4)
Generalized	125 (30.7)
Focal & generalized	5 (1.2)
Unknown	23 (5.7)
Epilepsy etiology	
Genetic^b^	224 (55.0)
Established	53 (13.0)
Presumed	171 (42.0)
Structural	82 (20.2)
Metabolic	7 (1.7)
Immune	0 (−)
Infectious	0 (−)
Unknown	94 (23.1)
Epilepsy syndrome	191 (46.9)
No epilepsy	737 (60.8)
Single unprovoked seizures^c^	38 (5.2)
Provoked seizures^d^	81 (11.0)
Cardiovascular events^e^	83 (11.3)
Vasovagal collapses	81 (11.0)
Cardiac events	2 (0.3)
Respiratory events	23 (3.1)
Behavioral events	250 (33.9)
Psychological & psychiatric conditions	32 (4.3)
Sleep‐related conditions	34 (4.6)
Paroxysmal movement disorders	35 (4.7)
Migraine associated disorders	27 (3.7)
Miscellaneous events	170 (23.1)
Unclear diagnosis	69 (5.7)

^a^ Data presented as *N* (%).

^b^ Genetic was subdivided into established genetic (known pathogenic mutation) and presumed genetic (e.g., probably pathogenic mutation, all idiopathic generalized epilepsies, self‐limited epilepsy with centrotemporal spikes).

^c^ Also includes children with ≥2 unprovoked seizures within 24 h, not meeting the ILAE definition of epilepsy.

^d^ Both febrile and acute symptomatic seizures (elicited by hypoglycemia, trauma, or medication).

^e^ From cardiovascular to miscellaneous events: events are not mutually exclusive. List of events derived from: International League Against Epilepsy (ILAE). Epilepsy imitators. EpilepsyDiagnosis.org: Diagnostic Manual. https://www.epilepsydiagnosis.org/epilepsy‐imitators.html. Updated July 15, 2022. Accessed April 5, 2023.

The median time to last follow‐up was 2.5 years (0 days to 13.6 years) for children with epilepsy, 0 days (0 days to 11.0 years) for children without epilepsy, and 3.5 months (0 days to 6.3 years) for children with an “unclear” final diagnosis.

#### 3.2.1 Children with epilepsy

Of the 407 children with a final “epilepsy” diagnosis, 271 (66.6%) received this diagnosis at the FSC consultation. Most of the remaining children had an “unclear” initial FSC diagnosis (84; 20.6%) or presented with a SUS (37; 9.1%). Fifteen children (3.7%) were initially misclassified as not having epilepsy (Table 3). Children with epilepsy were significantly older at their first SLE and at FSC consultation than those without epilepsy, and had shorter intervals between the SLE and FSC consultation (median 2.4 versus 3.6 months, *p* < 0.001). They also more often had a history of seizures and neurological disorders, specifically perinatal asphyxia, and were more likely to have developmental delay (Table S3).

**TABLE 3:** FSC and final diagnoses of all included children.

		Final diagnosis			
Initial FSC diagnosis		Epilepsy	No epilepsy^**b**^	Unclear	Total
	Epilepsy	271	6	2	279
	No epilepsy^a^	15	511^c^	0	526
	*Provoked seizure(s)*	*6*	*69*	*0*	*75*
	*Specified other diagnosis*	*0*	*325*	*0*	*325*
	*Unspecified other diagnosis*	*9*	*117*	*0*	*126*
	Single unprovoked seizure	37	33	1	71
	Unclear	84	187	66	337
	Total	407	737	69	1213

Abbreviation: FSC, first seizure clinic.

^a^ The no epilepsy diagnosis is subdivided into provoked seizure(s) (e.g., febrile seizures), specified other diagnosis (e.g., vasovagal collapse), and unspecified other diagnosis, that is: event(s) of unclear but non‐epileptic origin.

^b^ The final diagnosis of no epilepsy includes single unprovoked seizures without subsequent unprovoked seizures. Therefore, there is no category single unprovoked seizure category in the final diagnosis list.

^c^ After being diagnosed at the FSC (no epilepsy), doubts about the origin of the seizure‐like events arose after the FSC evaluation in 10 of these children. We regarded these children as being correctly diagnosed on the FSC evaluation day (time to diagnosis = 0).

Epileptiform abnormalities were seen in the FSC EEG recordings of 248 children with a final epilepsy diagnosis (60.9%), predominantly in those with a correct initial diagnosis (221/271, 81.5%) (Table S6). Also, epileptiform abnormalities were more frequently seen in childhood and adolescence compared to the neonatal and infantile age range (Table S4B). Almost a quarter of all children with epilepsy (94 children, 23.1%) had a normal EEG recording (Table 4). In 26 of them, epilepsy was nevertheless diagnosed at the FSC consultation. Their diagnoses were based on medical history (*N* = 7), family history (*N* = 10), and event description (*N* = 21) (not mutually exclusive). Seizures were captured on the FSC EEG recordings of 76 children (18.7%) (Table 4).

**TABLE 4:** FSC EEG recording results.

	Final diagnosis		
	Epilepsy (*N* = 407)^c^	No epilepsy (*N* = 737)^c^	Unclear (*N* = 69)^c^
Result^a^			
Normal EEG	94 (23.1)	588 (79.8)	52 (75.4)
Abnormal EEG^b^	311 (76.4)	145 (19.7)	17 (24.6)
Aspecific abnormalities	117 (28.7)	114 (15.5)	9 (13.0)
Epileptiform abnormalities	248 (60.9)	42 (5.7)	8 (11.6)
Focal epileptiform	150 (36.8)	34 (4.6)	7 (10.1)
Generalized epileptiform	42 (10.3)	5 (0.7)	0 (−)
Focal & generalized epileptiform	56 (13.8)	3 (0.4)	1 (1.5)
Events captured			
Seizures	76 (18.7)	0 (−)	0 (−)
Non‐epileptic events	4 (1.0)	56 (7.6)	0 (−)

Abbreviation: EEG, electroencephalogram.

^a^ Data presented as *N* (%).

^b^ Aspecific and epileptiform EEG abnormalities are not mutually exclusive.

^c^ Number of non‐assessable or absent EEGs: epilepsy = 2; no epilepsy = 4; unclear = 0.

Epilepsy type was focal in 254 (62.4%) and generalized in 125 (30.7%) children. A few children had a mixed (5; 1.2%) or unclear (23; 5.7%) epilepsy type (Table 2). Almost half of the children (191; 46.9%) were diagnosed with an epilepsy syndrome. Genetic generalized epilepsies and self‐limited epilepsy with centrotemporal spikes were the most prevalent syndromes, accounting for 44.0% and 24.1% of syndromes, respectively (Table S7). Epilepsy etiologies were presumed or established genetic (55.0%), structural (20.2%), metabolic (1.7%), and unknown (23.1%) (Table 2).

Between the FSC visit and the last follow‐up, 320 children (78.6%) were at some point treated with at least one ASM. At the last follow‐up, 230 of them were still using (one of) those. Sixteen simultaneously received other epilepsy treatments: ketogenic diet (*N* = 2), vagal nerve stimulation therapy (*N* = 4), and medication other than ASM (pyridoxine: *N* = 8; taurine: *N* = 2; cannabidiol: *N* = 1) (not mutually exclusive). Two children were on ketogenic diet without ASM. Twelve children underwent epilepsy surgery during follow‐up, with a mean time interval from FSC consultation to surgery of 1.6 years. At the last follow‐up, 163 children (40.1%) were seizure‐free, 165 (40.5%) were not, and seizure outcome was undetermined for 79 children (19.4%). Of all children with epilepsy, 310 (76.2%) were actively followed at our hospital for at least 1 year.

#### 3.2.2 Children without epilepsy

Of the 737 children without epilepsy, 511 (69.3%) were already diagnosed as such at the FSC consultation. Others initially had an unclear diagnosis (187; 25.4%) or presented with a SUS (33; 4.5%). Six children (0.8%) were initially erroneously diagnosed with epilepsy (Table 3).

The FSC EEG showed epileptiform abnormalities in 42 children (5.7%); approximately half of those (22/42) had an unclear initial FSC diagnosis (Table S6). In 10 children with epileptiform EEG abnormalities, the “epilepsy” diagnosis was nevertheless already rejected at the FSC consultation. Epileptiform EEG findings in these children were—in the absence of seizures—interpreted as rolandic spikes, seizure susceptibility, photosensitivity, or related to a structural brain abnormality or genetic mutation. Non‐epileptic events were captured on the FSC EEG recordings of 56 children (7.6%) (Table 4).

Etiological diagnoses in the “no epilepsy” group were diverse. Overall, behavioral events (33.9%; predominantly daydreaming or inattention), cardiovascular events (11.3%; generally reflex syncopes), and provoked seizures (11.0%; mostly febrile) were the most prevalent. Respiratory events (3.1%), migraine‐associated disorders (3.7%), and psychological and psychiatric conditions (4.3%) were the least common (Table 2). Psychogenic non‐epileptic seizures, that were included in the latter category, were diagnosed in nine children (1.2%). The prevalence of the distinct etiological diagnoses varied by age (Table S4C).

#### 3.2.3 Children with an unclear final diagnosis

Of the 69 children with an “unclear” final diagnosis, two (2.9%) were initially diagnosed with epilepsy and one (1.4%) with a SUS (Table 3). Most FSC EEG recordings were normal (52, 75.4%). Epileptiform abnormalities were seen in eight children (11.6%) (Table 4).

### 3.3 Diagnostic accuracy and time to diagnosis

**FIGURE 1:**
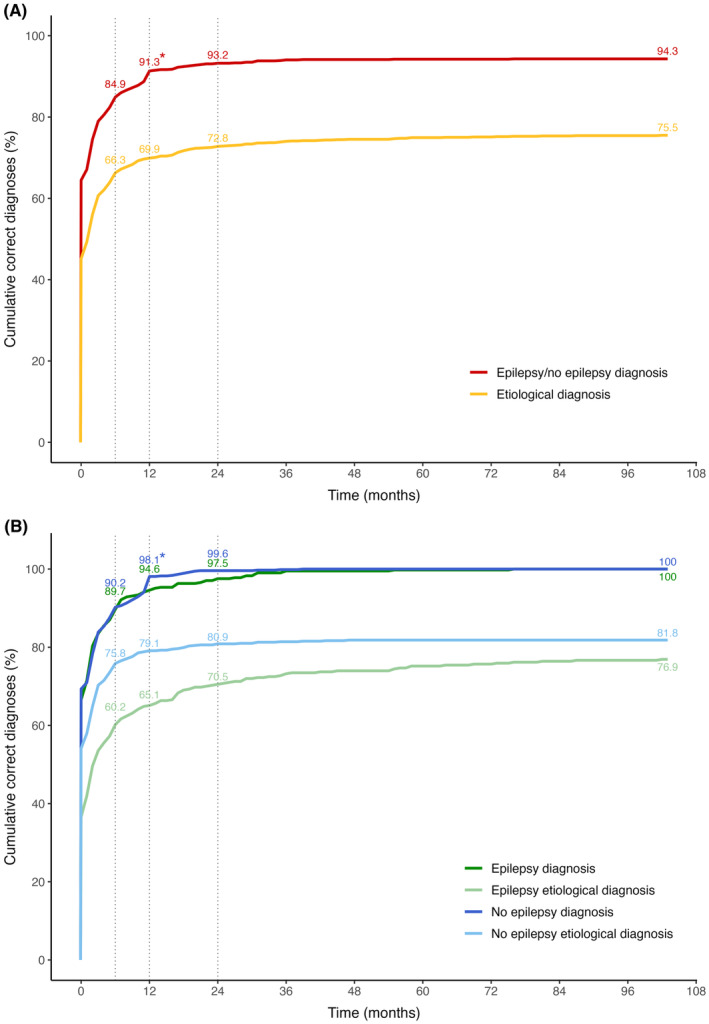
Time to diagnosis for all children together (A) and for children stratified by final diagnosis (B). Since the diagnosis remained unclear in 69/1213 children, the red line in panel A does not reach 100%. *Single unprovoked seizures were reclassified as no epilepsy 12 months post‐seizure, which causes a clear increase in the number of diagnoses around time = 12 months.

Of all children, 1144 (94.3%) received a final diagnosis of “epilepsy” or “no epilepsy.” In 782 of them (68.6%), the final diagnosis was already made at the FSC consultation. This number excludes the children with a SUS because their final diagnosis could only be made by follow‐up. Of all children with a clear initial FSC diagnosis of “epilepsy” or “no epilepsy” (*n* = 805), 23 (2.9%) were found to be misdiagnosed during follow‐up (Table 3). The mean time to final diagnosis (“epilepsy” or “no epilepsy”) was 2.0 months (range 0 days to 6.3 years); nearly 85% of all children were correctly diagnosed within 6 months and over 91% within 12 months (Figure 1).

## 4 DISCUSSION

We presented data from 1213 children who, over a 13‐year period, were referred to our FSC for the evaluation of SLEs. Our cohort is the largest to date and can serve as a clinical frame of reference for the distribution and specification of diagnoses in children presenting with new‐onset SLEs.

Ideally, all children evaluated for SLEs receive an immediate correct diagnosis. In our cohort, “epilepsy” or “no epilepsy” was correctly diagnosed at the FSC consultation in 68.6% of the children with such an eventual diagnosis. The misdiagnosis rate among those children was only 2.9%. This is considerably lower than previously reported misdiagnosis rates in children not (primarily) evaluated at an FSC.^3^, ^4^, ^5^, ^6^ Together with an Australian cohort wherein none of 200 children received an incorrect initial FSC diagnosis,^12^ these data underline an important potential diagnostic benefit of FSCs with consultation by experienced physicians. In contrast to other pediatric FSC cohorts,^6^, ^12^, ^13^ we report many children (27.8%) with an unclear initial FSC diagnosis. This may be explained by the diagnostic criteria used, since the initial FSC diagnosis of all children who underwent additional diagnostic investigations after the FSC evaluation was considered “unclear,” even if there was a working diagnosis of “epilepsy” or “no epilepsy.” In most children (55.5%) with an unclear initial FSC diagnosis, the epilepsy diagnosis was eventually rejected, highlighting our team's caution in rejecting an epilepsy diagnosis, although we were reluctant to treat patients with an unclear diagnosis with ASM. False‐negative diagnoses and, consequently, diagnostic and treatment delays may cause adverse outcomes.^19^, ^20^, ^21^

One‐third of the children (33.5%) were eventually diagnosed with epilepsy, and 42.0% presented with seizures. Other pediatric FSC studies reported epilepsy in 33.1%–54.5%^6^, ^12^ and epileptic events in 74.0%–76.2%.^6^, ^13^ Despite the variation across studies—most likely reflecting differences in study settings and referral policies—these numbers highlight how challenging it is for referrers to diagnose epilepsy and seizures. In contrast to previous studies, most children with epilepsy in our cohort had a follow‐up of multiple years (median 2.5 years). This allowed an in‐depth search for the underlying etiology, which was identified in 76.9% of all children. This is significantly higher than previously reported.^6^, ^22^, ^23^ A (presumed) genetic etiology accounted for 55.0%, representing our specific study population but also the rapid developments in the field of epilepsy genetics.^24^

EEG is a valuable tool for the evaluation of SLEs. The proportion of children with epileptiform EEG abnormalities reported after new‐onset seizures or epilepsy varies, however, widely (17.8%–63.7%)^6^, ^8^, ^9^, ^25^, ^26^, ^27^, ^28^ and may depend on the timing of the EEG recording.^8^ We found epileptiform abnormalities in the FSC EEG recording of 60.9% of children eventually diagnosed with epilepsy. This relatively high proportion probably represents the high prevalence (46.9% of children with epilepsy) of specific epilepsy syndromes in our cohort. Still, it indicates that in almost 40%, the final diagnosis was not supported by the FSC EEG findings. Additionally, not all children with a final epilepsy diagnosis who showed epileptiform abnormalities were diagnosed as such at the FSC, while conversely, some children without epileptiform abnormalities were. And epileptiform abnormalities were also found in 5.7% of children without epilepsy. These data emphasize the relevance of medical history, family history, and event description—accompanied by home videos—for determining the origin of SLEs.

The role of FSCs is broader than distinguishing epilepsy from its mimics and initiating treatment. The burden of comorbidity in children with epilepsy is high; additional medical, neurological, and developmental or psychiatric disorders are common.^29^ Early recognition of comorbidities is important as they might interact with or (help) guide epilepsy treatment and may require therapy, parent/patient education, and communication with school. FSCs are valuable here as awareness of comorbidities among experts contributes to early detection and the direct engagement of an epilepsy nurse—which is common practice at some FSCs—provides for timely education and support in dealing with practicalities in its broadest sense.

This study has limitations. Since data were collected retrospectively via medical records that were not designed to collect research data, there was missing data for a small number of children. Other biases, inherent to a retrospective study design, were limited by including all children that were evaluated at our FSC and the systematic approach of the FSC consultation. Our cohort may nevertheless be biased due to the relatively high number of internal referrals. Since our hospital is a tertiary children's hospital, many internally referred children were already diagnosed with any type of medical condition requiring specialist care. Although all FSCs aim at evaluating children with new‐onset SLEs, the specific study population being evaluated depends on geographics, referral policies, and triage processes. Using our cohort as a frame of reference should always be tailored to this.

An opportunity for future studies is to assess the (added) diagnostic value of additional investigations commonly performed in the diagnostic workup of SLEs, particularly in children with an unclear initial FSC diagnosis. In addition, the diagnostic accuracy of FSCs may benefit from incorporating prediction models or various artificial intelligence applications, which may help physicians better select and interpret information from medical and family history, event description, and EEG recordings.^30^, ^31^ This could lead to less treatment delay, fewer investigations, and reduced healthcare costs.

## 5 CONCLUSION

New‐onset SLEs are common in children. Since almost 60% of the referred children had not clearly experienced seizures and only one‐third were eventually diagnosed with epilepsy, this study shows the diagnostic challenges that referrers face in differentiating epilepsy from its mimics. In addition, the diagnostic value of epileptiform EEG abnormalities is moderate, highlighting the need for expertise. Nevertheless, our team correctly diagnosed almost 70% of children as early as the FSC consultation, and in less than 3% of children, an initial FSC “epilepsy” or “no epilepsy” diagnosis was later found to be incorrect. This enforces our belief that all children with SLEs should be evaluated by an experienced FSC team, preferably with rapidly available diagnostic investigations. This study—describing the largest pediatric FSC cohort to date—may serve as a clinical frame of reference for all physicians involved in the care of children with SLEs.

## AUTHOR CONTRIBUTIONS

Ms Geertruida Slinger conceptualized and designed the study, collected data, carried out the analyses, drafted the initial manuscript, and revised the manuscript; Ms Lotte Noorlag conceptualized and designed the study, collected data, drafted the initial manuscript, and revised the manuscript; Dr Eric van Diessen contributed in conceptualization and design of the study, and critically reviewed the manuscript; Dr Willem M. Otte contributed in conceptualization and design of the study, supervised the analyses, and critically reviewed the manuscript; Prof Maeike Zijlmans contributed in conceptualization and design of the study, and critically reviewed the manuscript; Dr Floor E. Jansen contributed in conceptualization and design of the study, and critically reviewed the manuscript; Prof Kees P.J. Braun contributed in conceptualization and design of the study, supervised the research process, and critically reviewed the manuscript; all authors approved the final manuscript as submitted and agree to be accountable for all aspects of the work.

## CONFLICT OF INTEREST STATEMENT

None of the authors has any conflicts of interest to disclose. We confirm that we have read the Journal's position on issues involved in ethical publication and affirm that this report is consistent with those guidelines.

## ETHICAL APPROVAL

The institutional ethics committee approved the use of anonymized retrospective data for research purposes without informed consent (protocol numbers 09‐353/K and 18‐354/C).

## Supporting information


Appendix S1.

## Data Availability

Research data are not shared.

## References

[1] Beghi E . The epidemiology of epilepsy. Neuroepidemiology. 2020;54(2):185–191. 10.1159/000503831 31852003

[2] International League Against Epilepsy (ILAE) . Epilepsy imitators. Accessed April 5, 2023. https://www.epilepsydiagnosis.org/epilepsy‐imitators.html

[3] Gibbs J , Appleton R . False diagnosis of epilepsy in children. Seizure. 1992;1(1):15–18. 10.1016/1059-1311(92)90049-7 1344314

[4] Stroink H , van Donselaar C , Geerts A , Peters A , Brouwer O , Arts W . The accuracy of the diagnosis of paroxysmal events in children. Neurology. 2003;60(6):979–982. 10.1212/01.wnl.0000049914.25434.72 12654963

[5] Uldall P , Alving J , Hansen L , Kibaek M , Buchholt J . The misdiagnosis of epilepsy in children admitted to a tertiary epilepsy Centre with paroxysmal events. Arch Dis Child. 2006;91(3):219–221. 10.1136/adc.2004.064477 16492886 PMC2065931

[6] Hamiwka L , Singh N , Niosi J , Wirrell E . Diagnostic inaccuracy in children referred with “first seizure”: role for a first seizure clinic. Epilepsia. 2007;48(6):1062–1066. 10.1111/j.1528-1167.2007.01018.x 17553117

[7] Shinnar S , Kang H , Berg A , Goldensohn E , Hauser W , Moshé S . EEG abnormalities in children with a first unprovoked seizure. Epilepsia. 1994;35(3):471–476. 10.1111/j.1528-1157.1994.tb02464.x 8026390

[8] King M , Newton M , Jackson G , Fitt GJ , Mitchell LA , Silvapulle MJ , et al. Epileptology of the first‐seizure presentation: a clinical, electroencephalographic, and magnetic resonance imaging study of 300 consecutive patients. Lancet. 1998;352(9133):1007–1011. 10.1016/S0140-6736(98)03543-0 9759742

[9] Baldin E , Hauser W , Buchhalter J , Hesdorffer D , Ottman R . Yield of epileptiform electroencephalogram abnormalities in incident unprovoked seizures: a population‐based study. Epilepsia. 2014;55(9):1389–1398. 10.1111/epi.12720 25041095 PMC4167205

[10] Grant A , Chau L , Arya K , Schneider M . Prevalence of epileptiform discharges in healthy 11‐ and 12‐year‐old children. Epilepsy Behav. 2016;62:53–56. 10.1016/j.yebeh.2016.06.020 27450305 PMC5014703

[11] Sorin L , Knupp K , Berg A . New‐onset seizure survey of epilepsy centers in the United States. Epilepsy Behav. 2019;101(Pt A):106579. 10.1016/j.yebeh.2019.106579 31677582

[12] Shah S , Nagarajan L , Palumbo L , Walsh P , Silberstein J , Cannell P , et al. Paediatric new‐onset seizure clinic in Australia: experience and lessons learnt. J Paediatr Child Health. 2019;55(7):789–794. 10.1111/jpc.14290 30407686

[13] Kim S , DeGrauw T , Berg A , Hass H , Koh S . Evaluation of pediatric patients in new‐onset seizure clinic (NOSc). Epilepsy Behav. 2020;112:107428. 10.1016/j.yebeh.2020.107428 32920376

[14] Fisher R , Acevedo C , Arzimanoglou A , Bogacz A , Cross JH , Elger CE , et al. ILAE official report: a practical clinical definition of epilepsy. Epilepsia. 2014;55(4):475–482. 10.1111/epi.12550 24730690

[15] Scheffer I , Berkovic S , Capovilla G , Connolly MB , French J , Guilhoto L , et al. ILAE classification of the epilepsies: position paper of the ILAE Commission for Classification and Terminology. Epilepsia. 2017;58(4):512–521. 10.1111/epi.13709 28276062 PMC5386840

[16] Wirrell E , Nabbout R , Scheffer I , Alsaadi T , Bogacz A , French JA , et al. Methodology for classification and definition of epilepsy syndromes with list of syndromes: report of the ILAE task force on nosology and definitions. Epilepsia. 2022;63(6):1333–1348. 10.1111/epi.17237 35503715

[17] Kwan P , Arzimanoglou A , Berg A , Brodie MJ , Allen Hauser W , et al. Definition of drug resistant epilepsy: consensus proposal by the ad hoc task force of the ILAE commission on therapeutic strategies. Epilepsia. 2010;51(6):1069–1077. 10.1111/j.1528-1167.2009.02397.x 19889013

[18] R Core Team . R: A language and environment for statistical computing. 2021.

[19] Berg AT , Loddenkemper T , Baca CB . Diagnostic delays in children with early onset epilepsy: impact, reasons, and opportunities to improve care. Epilepsia. 2014;55(1):123–132. 10.1111/epi.12479 24313635 PMC3946922

[20] Auvin S , Hartman AL , Desnous B , Moreau AC , Alberti C , Delanoe C , et al. Diagnosis delay in west syndrome: misdiagnosis and consequences. Eur J Pediatr. 2012;171(11):1695–1701. 10.1007/s00431-012-1813-6 22892960

[21] O'Callaghan F , Lux A , Darke K , Edwards SW , Hancock E , Johnson AL , et al. The effect of lead time to treatment and of age of onset on developmental outcome at 4 years in infantile spasms: evidence from the United Kingdom infantile spasms study. Epilepsia. 2011;52(7):1359–1364. 10.1111/j.1528-1167.2011.03127.x 21668442

[22] Sokka A , Olsen P , Kirjavainen J , Harju M , Keski‐Nisula L , Räisänen S , et al. Etiology, syndrome diagnosis, and cognition in childhood‐onset epilepsy: a population‐based study. Epilepsia Open. 2017;2(1):76–83. 10.1002/epi4.12036 29750215 PMC5939454

[23] Aaberg KM , Surén P , Søraas CL , Bakken IJ , Lossius MI , Stoltenberg C , et al. Seizures, syndromes, and etiologies in childhood epilepsy: the international league against epilepsy 1981, 1989, and 2017 classifications used in a population‐based cohort. Epilepsia. 2017;58(11):1880–1891. 10.1111/epi.13913 28949013

[24] Krey I , Platzer K , Esterhuizen A , Berkovic SF , Helbig I , Hildebrand MS , et al. Current practice in diagnostic genetic testing of the epilepsies. Epileptic Disord. 2022;24(5):765–786. 10.1684/epd.2022.1448 35830287 PMC10752379

[25] Scotoni AE , Manreza MLG , Guerreiro MM . Recurrence after a first unprovoked cryptogenic/idiopathic seizure in children: a prospective study from Sao Paulo, Brazil. Epilepsia. 2004;45(2):166–170. 10.1111/j.0013-9580.2004.16503.x 14738424

[26] Lizana JR , Garcia EC , Marina LLC , Lopez MV , Martin Gonzalez M , Hoyos AM . Seizure recurrence after a first unprovoked seizure in childhood: a prospective study. Epilepsia. 2000;41(8):1005–1013. 10.1111/j.1528-1157.2000.tb00286.x 10961628

[27] Sadleir LG , Scheffer IE . Optimizing electroencephalographic studies for epilepsy diagnosis in children with new‐onset seizures. Arch Neurol. 2010;67(11):1345–1349. 10.1001/archneurol.2010.155 20625070

[28] Tews W , Weise S , Syrbe S , Hirsch W , Viehweger A , Merkenschlager A , et al. Is there a predictive value of EEG and MRI after a first afebrile seizure in children? Klin Padiatr. 2014;227(2):84–88. 10.1055/s-0034-1394421 25419720

[29] Aaberg KM , Bakken IJ , Lossius MI , Lund Søraas C , Håberg SE , Stoltenberg C , et al. Comorbidity and childhood epilepsy: a Nationwide registry study. Pediatrics. 2016;138(3):e20160921. 10.1542/peds.2016-0921 27482059

[30] Van Diessen E , Lamberink HJ , Otte WM , Doornebal N , Brouwer OF , Jansen FE , et al. A prediction model to determine childhood epilepsy after 1 or more paroxysmal events. Pediatrics. 2018;142(6):e20180931. 10.1542/peds.2018-0931 30389715

[31] van Diessen E , Otte WM , Braun KP , Stam CJ , Jansen FE . Improved diagnosis in children with partial epilepsy using a multivariable prediction model based on EEG network characteristics. PloS One. 2013;8(4):e59764. 10.1371/journal.pone.0059764 23565166 PMC3614973

