# Supplementary material for: Clinical characteristics and diagnoses of 1213 children referred to a first seizure clinic
Source: Epilepsia Open. 2024 Jan 25;9(2):548–57. doi: 10.1002/epi4.12883 (PMC10984297; doi:10.1002/epi4.12883)
Supplement: Supplementary file 1 — Appendix S1. [file EPI4-9-548-s001.pdf]

Supplementary Material

Article title: Clinical characteristics and diagnoses of 1,213 children referred to a first seizure clinic

Supplementary Table 1. Definitions of variables

Variable	Definition
Demographics and medical history	
Sex	Biological sex of the child at birth. Options: boy; girl.
Age at FSLE	Age at which the FSLE (for which the child was evaluated at the FSC) occurred.
Age at FSC consultation	Age at which the child visited the FSC.
Referrer	Referrer of the child to the FSC. Options: general practitioner; internal specialist (Wilhelmina Children's Hospital); external specialist (another hospital or institute); self-referral.
Referral via ED	Referral of the child via the emergency department. Only applicable for children being referred by an internal or external specialist. Options: no; yes.
Family history	Relevant medical conditions in first- and second-degree relatives of the child. Options: no relevant medical conditions; epilepsy (any type); febrile seizures; migraine; other (consanguinity of parents, cerebrovascular accidents, or developmental delay)
History of epilepsy	Previous epilepsy diagnosis, with at the time of the FSC consultation seizure freedom, no ASM, and no ongoing follow-up. Options: no; yes.
History of seizures	Seizures other than in the context of epilepsy. Options: no; typical febrile seizures; atypical febrile seizures; neonatal convulsions; other acute symptomatic seizures.
Neurological history	History of neurological problems other than epilepsy or seizures. Options: no; perinatal asphyxia; hemorrhage or stroke; CNS infection; head trauma; migraine; other (e.g., known MCD, micro- or macrocephaly, neurological syndrome [due to genetic mutation]).
Neurodevelopmental disorder	Presence of a neurodevelopmental disorder, subdivided into before and/or after the FSC consultation. Options: none; AD(H)D; ASD.
Genetic syndrome	Presence of a genetic syndrome (including any mutation) at the time of the FSC consultation, including specific mutation. Options: none; yes, with specification.
Developmental concerns at presentation	Any developmental concerns at presentation, either being cognitive, motor, speech, social, emotional, or behavioral delay, mentioned by parent(s) or attending physician. Options: no; yes.
Intellectual disability	Any intellectual disability, either known or established at the time of the FSC visit or during follow-up; established or estimated IQ < 70. Options: no; yes.
Diagnosis	
Initial FSC diagnosis	Diagnosis made at the end of the FSC consultation. Options: epilepsy; no epilepsy; single unprovoked seizure; unclear.
Final diagnosis	Diagnosis at latest follow-up, if required after additional investigations. Options: epilepsy; no epilepsy (including single unprovoked seizures without subsequent unprovoked seizures in the twelve months following the FSC consultation); unclear.
Time to diagnosis	Time between the date of the FSC consultation and the date the final diagnosis was made. If the correct diagnosis was made on the day of the FSC consultation, the time to diagnosis was set to 0. In other cases, the time to diagnosis was calculated as the time between the date of the FSC consultation and the date that the epilepsy diagnosis was either confirmed or, alternatively, rejected.
Time to etiological diagnosis	Time between the date of the FSC consultation and the date the etiology of the epilepsy or the origin of the non-epileptic events became clear. This could not be calculated for children with an unclear final diagnosis, with epilepsy of unknown origin, with non-epileptic events of unclear origin, and with single unprovoked seizures (reclassified to no epilepsy after twelve months).
Final diagnosis epilepsy	
Epilepsy type	Epilepsy type. Options: focal; generalized; focal & generalized; unknown.
Epilepsy syndrome	Presence of an epilepsy syndrome according to the ILAE guidelines. [A]
Epilepsy etiology	Epilepsy etiology according to the ILAE guidelines. [B] Options: genetic; structural; metabolic; immune; infectious; unknown. Genetic was subdivided into established genetic (known pathogenic mutation) and presumed genetic (e.g., probably pathogenic mutation, all idiopathic generalized epilepsies, self-limited epilepsy with centrotemporal spikes).
Epilepsy surgery	Epilepsy surgery between the FSC consultation and last follow-up date. Options: no; yes.
ASM at last follow-up	Number of ASM used at last follow-up date.

Other treatment	Other treatment than ASM used at last follow-up date. Options: none; ketogenic diet; vagal nerve stimulator; other.
Seizure outcome	Seizure outcome at last follow-up date according to the ILAE guidelines. [C] Options: seizure-free; not seizure-free (or treatment failure); undetermined. If children only experienced one seizure, e.g., in the case of self-limited epilepsy with centrotemporal spikes or self-limited epilepsy with autonomic seizures from one seizure, we scored them as not seizure-free until twelve months after the single seizure.
Final diagnosis no epilepsy	
Etiology of final "no epilepsy" diagnosis	Etiology final "no epilepsy" diagnosis. Options: single unprovoked seizures (without subsequent unprovoked seizures in the 12 months following the FSC consultation); provoked seizures (both febrile and acute symptomatic seizures); cardiovascular events; respiratory events; behavioral events; psychological or psychiatric conditions; sleep-related conditions; paroxysmal movement disorders; migraine-associated disorders; miscellaneous events (including events not otherwise classifiable). For possible specifications, we refer to: "https://www.epilepsydiagnosis.org/epilepsy-imitators.html". [D]
FSC EEG results	
Results	Results of the EEG recording at the FSC consultation. Options: normal; aspecific abnormalities; epileptiform abnormalities, subdivided into focal, generalized, and focal & generalized epileptiform.
Events captured	Events captured during the EEG recording at the day of the FSC consultation. Options: none; seizures; non-epileptic events (only if these were the events that the child was evaluated for at the FSC).
Follow-up	
Last follow-up date	Date of the latest retrievable visit or phone consultation at the neurology department of our hospital (at the time of study inclusion).

AD(H)D: attention deficit (hyperactivity) disorder; ASD: autism spectrum disorder; ASM: anti-seizure medication; CNS: central nervous system; ED: emergency department; EEG: electroencephalogram; FSLE: first seizure-like event; FSC: first seizure clinic; ILAE: International League Against Epilepsy; IQ: intelligence quotient; MCD: malformation of cortical development

[A] Wirrell EC, Nabbout R, Scheffer IE, et al. Methodology for classification and definition of epilepsy syndromes with list of syndromes: Report of the ILAE Task Force on Nosology and Definitions. Epilepsia. 2022;63(6):1333-1348.

[B] Scheffer IE, Berkovic S, Capovilla G, et al. ILAE classification of the epilepsies: Position paper of the ILAE Commission for Classification and Terminology. Epilepsia. 2017;58(4):512-521.

[C] Kwan P, Arzimanoglou A, Berg AT, et al. Definition of drug resistant epilepsy: consensus proposal by the ad hoc Task Force of the ILAE Commission on Therapeutic Strategies. Epilepsia. 2010;51(6):1069-1077.

[D] International League Against Epilepsy (ILAE). Epilepsy imitators. EpilepsyDiagnosis.org: Diagnostic Manual. https://www.epilepsydiagnosis.org/epilepsy-imitators.html. Updated July 15, 2022. Accessed April 5, 2023.

Supplementary Table 2. Diagnostic rules concerning single unprovoked seizures and unclear diagnoses

FSC diagnosis	Final diagnosis	Diagnostic rule
Single unprovoked seizure	Epilepsy	Presentation with single unprovoked seizure AND (presence of subsequent unprovoked seizure[s] OR meeting the ILAE diagnostic criteria for epilepsy based on the outcomes of additional investigations performed after the FSC consultation)
Single unprovoked seizure	No epilepsy	Presentation with single unprovoked seizure AND (absence of subsequent unprovoked seizure[s] in the year following the first seizure OR no explicit representation in the year following the first seizure)
Single unprovoked seizure	Unclear	Presentation with single unprovoked seizure AND doubts about the epileptic origin of the first seizure based on follow-up and/or the outcomes of additional investigations performed after the FSC consultation
Unclear	Epilepsy	Presentation with event(s) of unclear origin AND (meeting the ILAE diagnostic criteria for epilepsy based on the outcomes of additional investigations performed after the FSC consultation OR diagnosis of epilepsy based on clinical presentation during follow-up)
Unclear	No epilepsy	Presentation with event(s) of unclear origin AND (rejection of the epilepsy diagnosis based on follow-up and/or the outcomes of additional investigations performed after the FSC consultation OR confirmation of an alternative non-epilepsy diagnosis after the FSC consultation)
Unclear	Unclear	Presentation with event(s) of unclear origin AND (inconclusive outcomes of additional investigations performed after the FSC consultation OR persistent contradiction between clinical presentation and the outcomes of additional investigations performed after the FSC consultation [e.g., clinical presentation suspicious for epilepsy, but additional investigations negative] OR lost to follow-up before additional investigations were performed OR discharged directly after the FSC consultation [with the note to return in case the frequency or semiology of the event changed])

EEG: electroencephalogram; FSC: first seizure clinic

Supplementary Table 3. Study population characteristics by final diagnosis

**Characteristic** [A]	**Epilepsy (N=407)**	**No epilepsy (N=737)**	**Unclear (N=69)**	**p-value** [B]
Demographics				
Sex, boy	230 (56.5)	398 (54.0)	40 (58.0)	0.42
Age at FSLE	7.1 (0-17.6) [C]	2.8 (0-17.3) [C]	2.4 (0.2-16.3) [C]	< 0.001
Age at FSC consultation	7.6 (0.1-17.7)	4.7 (0.2-17.4)	3.8 (0.3-17.3)	< 0.001
Family history [D, E, F]				
Epilepsy	77 (19.2)	151 (20.7)	17 (24.6)	0.59
Febrile seizures	38 (9.5)	88 (12.1)	4 (5.8)	0.20
Migraine	34 (8.5)	95 (13.0)	5 (7.2)	0.02
Other	41 (10.2)	49 (6.7)	10 (14.5)	0.04
Medical history [E]				
Epilepsy	14 (3.4)	14 (1.9)	0 (-)	0.11
Seizures [F]	77 (18.9)	77 (10.4)	9 (13.0)	< 0.001
Typical febrile seizures	49 (12.0)	46 (6.2)	6 (8.7)	0.001
Atypical febrile seizures	18 (4.4)	15 (2.0)	1 (1.4)	0.03
Neonatal convulsions	21 (5.2)	15 (2.0)	2 (2.9)	0.01
Other	5 (1.2)	10 (1.4)	0 (-)	1
Neurological [F]	93 (22.9)	128 (17.4)	12 (17.4)	0.03
Perinatal asphyxia	20 (4.9)	8 (1.1)	2 (2.9)	< 0.001
Hemorrhage or stroke	28 (6.9)	34 (4.6)	4 (5.8)	0.13
CNS infection	8 (2.0)	10 (1.4)	0 (-)	0.46
Head trauma	19 (4.7)	44 (6.0)	1 (1.4)	0.42
Migraine	4 (1.0)	7 (0.9)	0 (-)	1
Other	40 (9.8)	49 (6.6)	6 (8.7)	0.07
Neurodevelopmental disorder [F]	48 (11.8)	93 (12.6)	5 (7.2)	0.71
AD(H)D	18 (4.4)	40 (5.4)	1 (1.4)	0.49
ASD	38 (9.3)	64 (8.7)	4 (5.8)	0.75
Genetic syndrome or condition	37 (9.1)	50 (6.8)	11 (15.9)	0.16
Developmental status				
Developmental concerns at presentation [G]	157 (38.6)	240 (32.6)	29 (42.0)	0.04
Intellectual disability [H]	93 (22.9)	78 (10.6)	10 (14.5)	< 0.001

AD(H)D: attention deficit (hyperactivity) disorder; ASD: autism spectrum disorder; CNS: central nervous system; FSLE: first seizure-like event; FSC: first seizure clinic

[A] N (%) for categorical variables, and median (range) for continuous variables

[B] p-value for comparison between epilepsy and no epilepsy group; no comparisons made with the unclear group

[C] Data based on 385, 639, and 63 observations for the epilepsy, no epilepsy, and unclear group, respectively

[D] Family history in first- and second-degree relatives; data based on 401, 730, and 69 observations, for the epilepsy, no epilepsy, and unclear group, respectively

[E] Known at the time of the FSC consultation

[F] Subcategories of family history, seizures, neurological history, and neurodevelopmental disorders are not mutually exclusive

[G] Any, either being cognitive, motor, language/speech, social, emotional, or behavioral delay, mentioned by parent(s) or attending physician

[H] Established or estimated IQ <70, either known at the time of the FSC consultation or during follow-up

Supplementary Table 4A. Demographics and final diagnosis by age category

	**Neonatal and infancy** (≤ 2 years)	**Childhood** (>2 & ≤ 12 years)	**Adolescence** (>12 years)
Number of children	382	678	153
Demographics & medical history			
Sex, boy	204 (53.4)	383 (56.5)	81 (52.9)
Seizures	34 (8.9)	110 (16.2)	19 (12.4)
Neurological event or disorder	62 (16.2)	132 (19.5)	39 (25.5)
Neurodevelopmental disorder	14 (3.7)	101 (14.9)	31 (20.3)
Genetic syndrome or condition	27 (7.1)	61 (9.0)	10 (6.5)
Final diagnosis			
Epilepsy	64 (16.8)	274 (40.4)	69 (45.1)
No epilepsy	289 (75.7)	370 (54.6)	78 (51.0)
Unclear	29 (7.6)	34 (5.0)	6 (3.9)

Supplementary Table 4B. FSC EEG recording results and diagnosis specification for children with epilepsy by age category

	**Neonatal and infancy** (≤ 2 years)	**Childhood** (>2 & ≤ 12 years)	**Adolescence** (>12 years)
Number of children	64	274	69
FSC EEG recording [A]			
Normal EEG	32 (50.0)	48 (17.5)	14 (20.3)
Abnormal EEG [B]	32 (50.0)	224 (81.8)	55 (79.7)
Aspecific abnormalities	13 (20.3)	77 (28.1)	27 (39.1)
Epileptiform abnormalities	26 (40.6)	182 (66.4)	40 (58.0)
Focal epileptiform	16 (25.0)	111 (40.5)	23 (33.3)
Generalized epileptiform	5 (7.8)	28 (10.2)	9 (13.0)
Focal & generalized epileptiform	5 (7.8)	43 (15.7)	8 (11.6)
Diagnosis specification			
Epilepsy type			
Focal	37 (57.8)	182 (66.4)	35 (50.7)
Generalized	17 (26.6)	78 (28.5)	30 (43.5)
Focal & generalized	4 (6.2)	1 (0.4)	0 (-)
Unknown	6 (9.4)	13 (4.7)	4 (5.8)
Epilepsy etiology			
Genetic [C]	25 (39.0)	164 (59.9)	35 (50.7)
Established	11 (17.2)	37 (13.5)	5 (7.2)
Presumed	14 (21.9)	127 (46.4)	30 (43.5)
Structural	14 (21.9)	50 (18.2)	18 (26.1)
Metabolic	3 (4.6)	2 (0.7)	2 (2.9)
Immune	0 (-)	0 (-)	0 (-)
Infectious	0 (-)	0 (-)	0 (-)
Unknown	22 (35.5)	58 (21.2)	14 (20.3)
Epilepsy syndrome	22 (35.5)	137 (50.0)	32 (46.4)

Supplementary Table 4C. FSC EEG recording results and diagnosis specification for children without epilepsy by age category

	**Neonatal and infancy** (≤ 2 years)	**Childhood** (>2 & ≤ 12 years)	**Adolescence** (>12 years)
Number of children	289	370	78
FSC EEG recording [A]			
Normal EEG	259 (89.6)	283 (76.5)	46 (59.0)
Abnormal EEG [B]	28 (9.7)	86 (23.2)	31 (39.7)
Aspecific abnormalities	22 (7.6)	64 (17.3)	28 (35.9)
Epileptiform abnormalities	9 (3.1)	30 (8.1)	3 (3.8)
Focal epileptiform	8 (2.8)	23 (6.2)	3 (3.8)
Generalized epileptiform	1 (0.3)	4 (1.1)	0 (-)
Focal & generalized epileptiform	0 (-)	3 (0.8)	0 (-)
Diagnosis specification			
Single unprovoked seizures [D]	4 (1.4)	24 (6.5)	10 (12.8)
Provoked seizures [E]	48 (16.6)	32 (8.6)	1 (1.3)
Cardiovascular events [F]	25 (8.7)	39 (10.5)	19 (24.4)
Vasovagal collapses	23 (8.0)	39 (10.5)	19 (24.4)
Cardiac events	2 (0.7)	0 (-)	0 (-)
Respiratory events	13 (4.5)	6 (1.6)	4 (5.1)
Behavioral events	100 (34.6)	141 (38.1)	9 (11.5)
Psychological & psychiatric conditions	2 (0.7)	14 (3.8)	16 (20.5)
Sleep related conditions	9 (3.1)	23 (6.2)	2 (2.6)
Paroxysmal movement disorders	16 (5.5)	17 (4.6)	2 (2.6)
Migraine associated disorders	3 (1.0)	17 (4.6)	7 (9.0)
Miscellaneous events	80 (27.7)	74 (20)	16 (20.5)

Supplementary Table 4D. FSC EEG recording results and diagnosis specification for children with an unclear final diagnosis by age category

	**Neonatal and infancy** (≤ 2 years)	**Childhood** (>2 & ≤ 12 years)	**Adolescence** (>12 years)
Number of children	29	34	6
FSC EEG recording [A]			
Normal EEG	25 (86.2)	22 (64.7)	5 (83.3)
Abnormal EEG [B]	4 (13.8)	12 (35.3)	1 (16.7)
Aspecific abnormalities	4 (13.8)	4 (11.8)	1 (16.7)
Epileptiform abnormalities	0 (-)	8 (23.5)	0 (-)
Focal epileptiform	0 (-)	7 (20.6)	0 (-)
Generalized epileptiform	0 (-)	0 (-)	0 (-)
Focal & generalized epileptiform	0 (-)	1 (2.9)	0 (-)

Data are presented as N or N (%). EEG: electroencephalogram; FSC: first seizure clinic

[A] Number of non-assessable or absent EEGs (final diagnosis/age category): epilepsy/childhood = 2; no epilepsy/neonatal and infancy: 2; no epilepsy/childhood: 1; no epilepsy/adolescence: 1

[B] Aspecific and epileptiform EEG abnormalities are not mutually exclusive

[C] Genetic was subdivided into established genetic (known pathogenic mutation) and presumed genetic (e.g., probably pathogenic mutation, all idiopathic generalized epilepsies, self-limited epilepsy with centrotemporal spikes)

[D] Also includes children with ≥2 unprovoked seizures within 24 hours, not meeting the ILAE definition of epilepsy

[E] Both febrile and acute symptomatic seizures (elicited by hypoglycemia, trauma or medication)

[F] From cardiovascular to miscellaneous events: events are not mutually exclusive. List of events derived from: International League Against Epilepsy (ILAE). Epilepsy imitators. EpilepsyDiagnosis.org: Diagnostic Manual. https://www.epilepsydiagnosis.org/epilepsy-imitators.html. Updated July 15, 2022. Accessed April 5, 2023.

Supplementary Table 5. Referrers and final diagnoses per referrer

	Referrer				
**Final diagnosis**	General practitioner (N=519)	Internal specialist (N=448)	External specialist (N=224)	Self-referral (N=13)	Unknown (N=9)
Epilepsy	165 (31.8)	161 (35.9)	74 (33.0)	3 (23.1)	4 (44.4)
No epilepsy	331 (63.8)	255 (56.9)	136 (60.7)	10 (76.9)	5 (55.6)
Unclear	23 (4.4)	32 (7.2)	14 (6.3)	0 (-)	0 (-)

Data are presented as N (%)

Supplementary Table 6. FSC EEG recording results by initial FSC and final diagnosis

	Final diagnosis											
	Epilepsy (N=407) [C]				No epilepsy (N=737) [C]				Unclear (N=69) [C]			
	Initial FSC diagnosis											
	Epilepsy (N=271)	No epilepsy (N=15)	SUS (N=37)	Unclear (N=84)	Epilepsy (N=6)	No epilepsy (N=511)	SUS (N=33)	Unclear (N=187)	Epileps y (N=2)	No epilepsy (N=0)	SUS (N=1)	Unclear (N=66)
Result [A]												
Normal EEG	26 (9.6)	10 (66.7)	22 (69.5)	36 (42.9)	3 (50.0)	443 (86.7)	15 (45.5)	127 (67.9)	1 (50)	-	1 (100)	50 (75.8)
Abnormal EEG [B]	244 (90.0)	5 (33.3)	15 (40.5)	47 (56.0)	3 (50.0)	67 (13.1)	16 (48.5)	59 (31.6)	1 (50)	-	0 (-)	16 (24.2)
Aspecific abnormalities	68 (25.1)	4 (26.7)	11 (29.7)	34 (40.5)	0 (-)	62 (12.1)	10 (30.3)	42 (22.5)	1 (50)	-	0 (-)	8 12.1)
Epileptiform abnormalities	221 (81.5)	1 (6.7)	5 (13.2)	21 (25.0)	3 (50.0)	10 (2.0)	7 (21.2)	22 (11.8)	0 (-)	-	0 (-)	8 (12.1)
Focal epileptiform	129 (47.6)	1 (6.7)	5 (13.2)	15 (17.9)	2 (33.3)	9 (1.8)	7 (21.2)	16 (8.6)	0 (-)	-	0 (-)	7 (10.6)
Generalized epileptiform	38 (14.0)	0 (-)	0 (-)	4 (4.8)	0 (-)	1 (0.2)	0 (-)	4 (2.1)	0 (-)	-	0 (-)	0 (-)
Focal & generalized epileptiform	54 (19.9)	0 (-)	0 (-)	2 (2.4)	1 (16.7)	0 (-)	0 (-)	2 (1.1)	0 (-)	-	0 (-)	1 (1.5)
Events captured												
Seizures	76 (28.0)	0 (-)	0 (-)	0 (-)	0 (-)	0 (-)	0 (-)	0 (-)	0 (-)	-	0 (-)	0 (-)
Non-epileptic events	0 (-)	1 (6.7)	1 (2.7)	2 (2.4)	0 (-)	47 (9.2)	0 (-)	9 (4.8)	0 (-)	-	0 (-)	0 (-)

EEG: electroencephalogram; FSC: first seizure clinic; SUS: single unprovoked seizure

[A] Data presented as N (%)

[B] Aspecific and epileptiform EEG abnormalities are not mutually exclusive

[C] Number of non-assessable or absent EEGs (final diagnosis/FSC diagnosis): epilepsy/epilepsy = 1; epilepsy/unclear = 1; no epilepsy/no epilepsy = 1; no epilepsy/SUS = 2; no epilepsy/unclear = 1

Supplementary Table 7. Overview of epilepsy syndromes

**Epilepsy syndrome**	**Total (N=191)**
Neonatal and infantile	
Self-limited (familial) infantile epilepsy	5
Infantile epileptic spasms syndrome	5
(Genetic) epilepsy with febrile seizures plus	5
Myoclonic epilepsy in infancy	3
Dravet syndrome	2
Self-limited (familial) neonatal epilepsy	1
Childhood	
Self-limited epilepsy with centrotemporal spikes	46
Childhood absence epilepsy	26 [A]
Self-limited epilepsy with autonomic seizures	17
Atypical self-limited epilepsy with centrotemporal spikes	4
(Developmental and) epileptic encephalopathy with spike-and-wave activation in sleep	4
Epilepsy with myoclonic absences	4
Epilepsy with eyelid myoclonia	3
Epilepsy with myoclonic-atonic seizures	1
Childhood occipital visual epilepsy	1
Sleep-related hypermotor (hyperkinetic) epilepsy	1
Adolescence and adulthood	
Juvenile myoclonic epilepsy	22
Juvenile absence epilepsy	15 [B]
Variable age	
Familial focal epilepsy with variable foci	2
Epilepsy with auditory features	1
Other	
Genetic generalized epilepsy	21 [C]
Infantile epilepsy syndrome (not further specified)	1
Rett-like syndrome	1

[A] One child was later diagnosed with juvenile absence epilepsy, and another with juvenile myoclonic epilepsy

[B] One child was later diagnosed with juvenile myoclonic epilepsy

[C] Not fulfilling the criteria for one of the four idiopathic generalized epilepsies

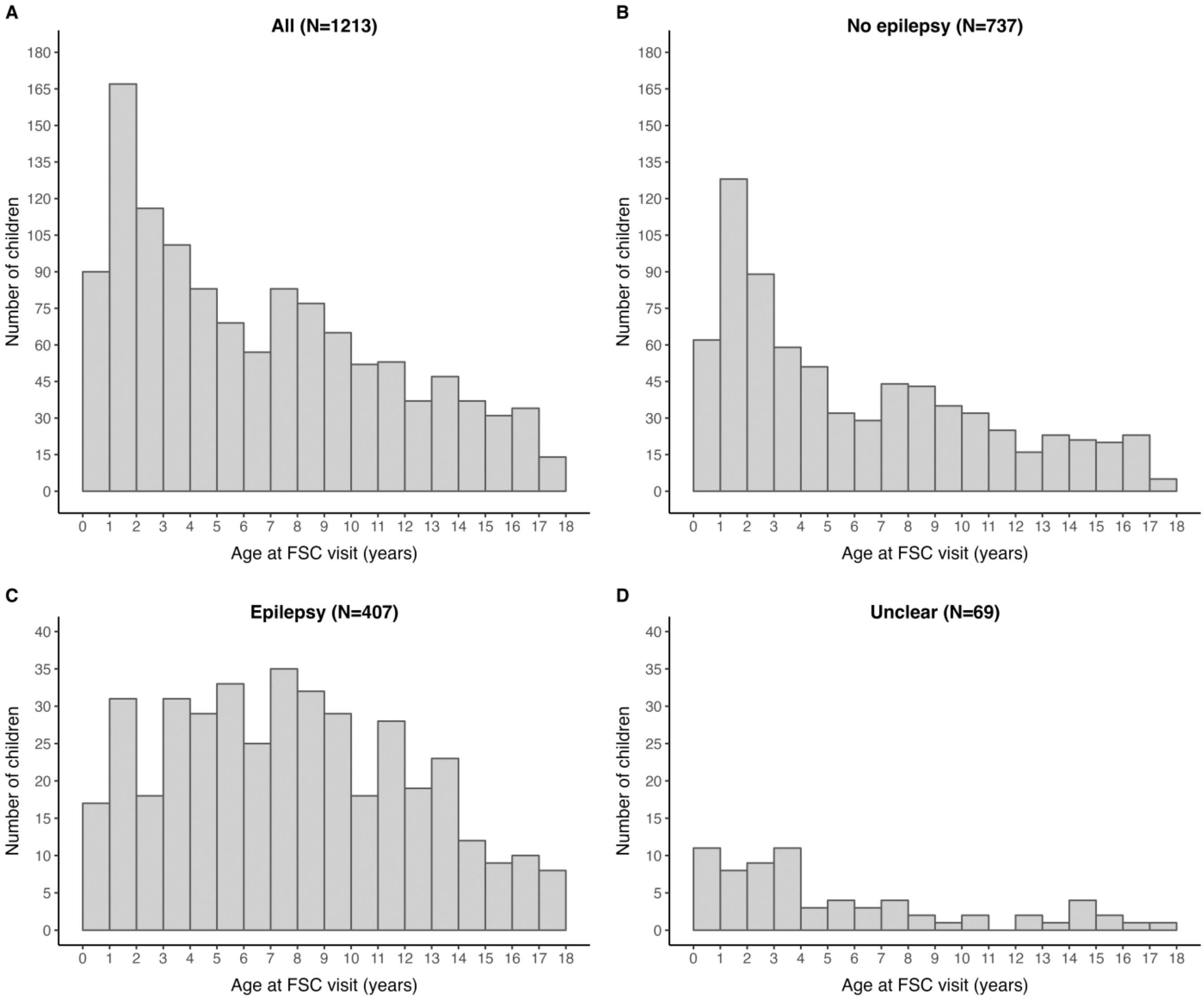

Supplementary Figure 1. Distribution of age at FSC visit for all children together (A), children without epilepsy (B), children with epilepsy (C), and children with an unclear final diagnosis (D)
